# A new perspective of hypothalamic disease: Shapiro's syndrome

**DOI:** 10.3389/fneur.2022.911332

**Published:** 2022-07-29

**Authors:** Linan Ren, Xiaokun Gang, Shuo Yang, Meixin Sun, Guixia Wang

**Affiliations:** ^1^Department of Endocrinology and Metabolism, The First Hospital of Jilin University, Changchun, China; ^2^Department of Clinical Nutrition, The First Hospital of Jilin University, Changchun, China

**Keywords:** Shapiro's syndrome, hypothermia, hyperhidrosis, agenesis of the corpus callosum, hypothalamic dysfunctional

## Abstract

Shapiro's syndrome (SS) is characterized by spontaneous periodic hypothermia. It occurs to patients regardless of age or sex. To date, <60 cases have been reported worldwide. Current knowledge of the disease is limited to clinical feature since the pathogenesis and etiology are still controversial. In this review, the clinical characteristics, pathological mechanism, and possible etiology of the syndrome were reviewed to improve the clinical understanding of the disease.

## Introduction

Shapiro's syndrome (SS), traditionally defined as the triad of spontaneous periodic hypothermia, hyperhidrosis, and agenesis of the corpus callosum (ACC), is a rare disease (1). However, spontaneous periodic hypothermia has been reported in recent years as a hallmark of both typical and variant SS (2). To date, <60 cases have been reported worldwide (3). The limited amount of data may be related to a lack of awareness among clinicians about the disease. Most of the publications on this disease were case reports, and only a limited number of reviews were based on observational studies of its clinical manifestations. The pathological mechanism of SS is still unclear, and there are several hypotheses that are hypothalamic dysfunction, neurotransmitter disorder (4–6), endogenous high melatonin, and genetic variation (2). This review summarizes the clinical manifestations and pathological mechanisms of the syndrome in order to improve clinicians' understanding of the disease.

## Overview of clinical presentation

SS was first reported in 1969 and was characterized by hypothermia and hyperhidrosis associated with agenesis of the corpus callosum (1). In 1994, patients with SS with an intact corpus callosum were reported and considered to be a variant of SS (7). To date, about 60 cases have been reported worldwide, including adults and children. This paper summarizes the current available cases of SS, with the exception of some cases where complete patient information is not available (Table 1) (3, 4, 7–12, 14–22, 27–29, 33–37). These results suggest that spontaneous periodic hypothermia is a hallmark of typical and variant SS, and that hyperhidrosis and ACC are supportive features not observed in all cases, consistent with a previous finding (2). Moreover, we found that the clinical manifestations of the syndrome included autonomic nervous dysfunction, complications related to hypothermia, and complications related to agenesis of the corpus callosum, etc. (see Table 2 for details).

**Table 1:** Articles and characteristics of patients with Shapiro's syndrome.

**Category**	**Clinical features**		**Number** **(*****n*** = **50)**	**References**
Basic Features	Age (years)	<20	25 (50%)	(2, 5–13)
		20–50	19 (38%)	(1, 3, 4, 13–26)
		>50	6 (12%)	(27–32)
	Gender	Female	25 (50%)	(1, 5–10, 12–15, 19, 20, 25, 27, 28, 30–32)
		Male	25 (50%)	(2–4, 7, 8, 11, 13, 16–18, 21–24, 26, 29)
	Duration (years)	≤ 10	33 (66%)	(2, 3, 5–13, 15, 16, 24, 25, 27, 31)
		>10	9 (18%)	(1, 4, 14, 17, 19, 22, 23, 30, 32)
	Attack Time (hours)	<1h	13 (26%)	(1, 7, 8, 11, 13, 19, 20, 26, 29–31)
		1–3h	10 (20%)	(1–3, 5, 7, 9, 12, 13, 21, 22, 32)
		3–6h	14 (28%)	(6, 8, 10)
		>6h	5 (10%)	(7, 15, 17, 24, 27)
Symptoms	Hypothermia		50 (100%)	(1–32)
	Hyperhidrosis		44 (88%)	(1–14, 16, 17, 19–23, 25–32)
	Flush		9 (18%)	(1, 3, 8, 17, 21, 22, 28)
	Pallor		20 (40%)	(2, 5–8, 10–13, 27)
	Chill		20 (40%)	(1, 2, 4–6, 8, 10, 13, 16, 17, 19, 21, 22, 24, 28, 31)
	Feelinghot		4 (8%)	(7, 23, 30, 32)
	Fatigue		8 (16%)	(1, 4, 8, 14)
	Headache		8 (16%)	(8, 21, 27)
	Faint		4 (8%)	(5, 6, 10, 13)
	Unresponsive		5 (10%)	(1, 7, 12, 21, 23)
	Alteredconsciousness		24 (48%)	(1, 4–10, 12, 13, 15–17, 20, 24, 28, 30)
	Cognitive impairment		3 (6%)	(7, 27)
	Urinaryincontinence		3 (6%)	(1, 6, 23)
	Urgency to urinate		2 (4%)	(6, 30)
	Sleep disorder		5 (10%)	(8, 14)
	Hallucinations		3 (6%)	(8)
	Anxietyordepression		3 (6%)	(14, 17, 31)
	Mental disorder		5 (10%)	(13, 16, 18, 28, 32)
	Bradycardia		20 (40%)	(1, 4, 7, 8, 12, 13, 17, 18, 21, 23–25, 28)
	Hypotension		10 (20%)	(1, 8, 15, 17, 23, 25, 29, 31)
	Dyspnea		4 (8%)	(1, 2, 7, 24)
	Abdominalpain		6 (12%)	(6–8, 12)
	Vomiting		4 (8%)	(8, 12, 28)
	Incoordination		4 (8%)	(8, 18, 20, 23)
	Dehydration		3 (6%)	(8, 16, 24)
	Tinnitus		1 (2%)	(1)
Laboratory and imaging features	Anemia		4 (8%)	(1, 15, 23, 24)
*(Continued)*				
**Category**	**Clinical features**		**Number** **(*****n*** = **50)**	**References**
	Leucopenia		1 (2%)	(23)
	Thrombocytopenia		3 (6%)	(15, 23, 24)
Laboratory and imaging features	Hypothyroidism		3 (6%)	(1, 11, 30)
	Decreasedsexhormones		3 (6%)	(4, 21, 30)
	Growth hormone deficiency		1 (2%)	(24)
	Hyponatremia		4 (8%)	(15, 23, 25, 31)
	Decrease in HVA and 5-HIAA in CSF		2 (4%)	(5)
	Endogenous hypermelatonemia		1 (2%)	(6)
	ACC		25 (50%)	(1, 3, 4, 8, 13–17, 19, 21–26, 28–32)
	SPECT: increased perfusion in regions near the thalamus		2 (4%)	(6, 10)
	FDG-PET:Increased metabolism in regions near the thalamus		1 (2%)	(28)

*Based on the actual number, the percentage is for reference, especially in the symptoms, laboratory, and imaging examination sections. ACC, agenesis of the corpus callosum; HVA, homovanillic acid; 5-HIAA, 5-hydroxyindoleacetic acid; CSF, cerebrospinal fluid; SPECT, single-photon emission computed tomography; FDG-PET,18F-fluorodeoxyglucose positron emission tomography*.

**Table 2:** Clinical features of Shapiro's syndrome.

**Category**	**Clinical features**
Autonomic nervous dysfunction	Hypothermia, hyperhidrosis, hypotension, dyspnea bradycardia, flush, pallor, abdominal pain, vomiting, headache, sleep disorder, urinary incontinence, urgency to urinate
Complications related to hypothermia	Unresponsive, altered consciousness, chills, shiver, edema, anemia, thrombocytopenia, leukopenia
Complications related to ACC	Epilepsy, cognitive impairment, physical dysplasia, incoordination or ataxia
Undetermined symptoms	Fatigue, tinnitus, depression or anxiety, hallucination, mental disorder, dehydration

## The role of the hypothalamus in Shapiro's syndrome

The pathophysiological mechanism of SS is still controversial. Initially, William Shapiro et al. (1) considered the syndrome to be “diencephalic epilepsy,” but this hypothesis was contradicted by the variant form of SS and the non-epileptiform focal changes of the EEG (17, 23). At present, there are three hypotheses, which are hypothalamic dysfunction, neurotransmitter dysfunction (2, 5, 9, 20, 27), and endogenous hypermelatonemia (6).

### Hypothalamic dysfunction

The hypothalamus is the body's thermo regulatory center. It mainly functions on the anterior preoptic area, the anterior center controls heat dissipation by inducing vasodilatation and sweating, and the posterior center conserves heat by inducing vasoconstriction and shivering. Dysfunction of the anterior center may result in fever, and dysfunction of the posterior center may cause hypothermia. At the same time, the hypothalamus is also the neuroendocrine regulation center. Symptoms of autonomic dysfunction occur when the cortical and hippocampal hypothalamus fibers and afferent fibers from the septum to the hypothalamus are destroyed (24, 30). These theories provide a reasonable explanation for the clinical manifestations of SS. Previously, Noel et al. (17) have found severe neuronal loss and fibroglial proliferation in the infundibular nucleus of hypothalamus, especially in the arcuate nucleus, in the postmortem pathological examination of a patient with SS. Similarly, Pineda et al. (36) reported that moderate spongiosis was found in the anterior and the lateral hypothalamic nuclei in two cases of agenesis of the corpus callosum with hypothermia. These findings confirm that hypothalamic lesions play an important role in SS pathogenesis. However, patients with SS syndrome, especially those with SS variant (SS with intact corpus callosum), showed normal brain structural imaging by magnetic resonance imaging (MRI) and other imaging examinations. In recent years, functional neuroimaging data of SS have been found. Dundar et al. (10) utilized technetium 99 m-labeled hexamethylpropylene amine oxime single-photon emission computed tomography (SPECT) in a patient with SS variant, which found increased perfusion in the right thalamus, basal ganglia, and inferior frontal areas. Pazderska et al. (28) employed (18) F-fluorodeoxyglucose positron emission tomography (FDG-PET) in a patient with SS variant, and reported mild increases in metabolism in the tectal plate regions bilaterally, posterior pons, posterior medulla, and a superior margin of the cerebellar vermis. Clearly, these areas of increased activity have been shown to be involved in thermoregulation, such as the right thalamus posterior pons and the medulla (38, 39). It is plausible that the hyperperfusion and hypermetabolism may be a secondary or compensatory response to hypothermia rather than direct evidence of hypothalamic lesions. Together, the essence of SS is hypothalamic dysfunction; however, there is a lack of reliable examination for the diagnosis of patients with SS.

### Hypermelatonemia

Melatonin is a hormone, which is recognized as the regulator of sleep-wake cycles, secreted primarily by the pineal gland. It is reported that melatonin played a role in the modulation of arterial blood pressure, locomotion, and thermoregulation (40). The result of a systematic review demonstrated that hypothermia was one of the adverse events in melatonin-treated sleep disorders (41). In a case of SS with hypermelatonemia, Duman et al. (6) found that the serum melatonin level increased markedly at midnight, and her symptoms were aggravated. It suggested the association between hypermelatonemia and the development of SS. However, this was the only reported case of SS with hypermelatonemia (6). Whether all patients with SS suffer from hypermelatonemia will need further clinical confirmation. Additionally, the rhythmic secretion of melatonin is regulated by the suprachiasmatic nucleus of the hypothalamus (42). Studies have shown that melatonin levels were significantly correlated with hypothalamic gray matter volume and disease severity in Parkinson's disease (PD) (43). Thus, we support the hypothesis of Duman et al. (6) that hypermelatonemia was secondary to hypothalamic dysfunction.

### Neurotransmitter dysregulation

Patients with SS had obvious symptoms of autonomic nervous dysfunction. Previous studies have confirmed that neurotransmitter disorders, such as dopamine and serotonin, are associated with the development of SS (19, 31, 44). Based on “threshold temperature for shivering” and the patient response to cyproheptadine, Sheth et al. (7) reported that specific serotonin dysfunction in the extrapyramidal shivering mechanism in the anterior hypothalamus is central to the pathogenesis of paroxysmal spontaneous hypothermia with hyperhidrosis. Increased plasma levels of norepinephrine have been reported in a patient with SS (31). Rodrigues et al. (5) reported two cases of SS, which showed reduced metabolites of 5-HIAA, HVA, 5-hydroxytryptamine and dopamine in CSF. Recently, drugs-regulating neurotransmitters, such as clonidine, cyproheptadine, pizotifen, and chlorpromazine, have achieved certain efficacy in the treatment of SS (2, 9, 20, 27, 31, 44). These results suggest that neurotransmitter regulation plays a role in SS pathogenesis. However, as mentioning above, the hypothalamus plays an important role in neuroregulation. Moreover, results of Rodrigues et al. were challenged by Duman et al. (45) who reported a case of SS in which 5-HIAA, vanillylmandelic, and HVA were normal in urine at 24 h. Therefore, we hypothesized that neurotransmitter dysregulation plays a secondary role in SS.

**Figure 1:**
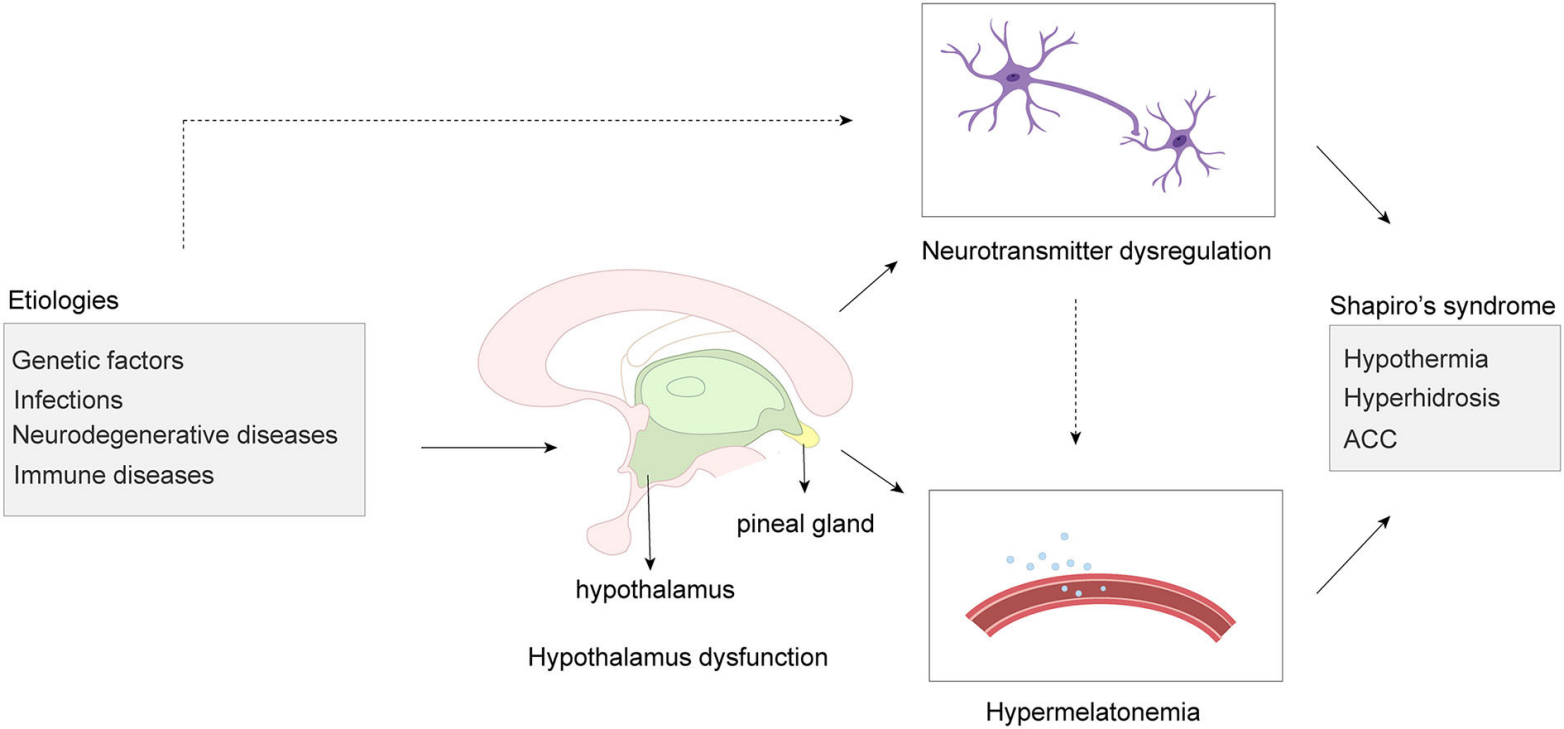
Schematic illustration of the etiologies and pathological mechanisms of the Shapiro syndrome. The dashed arrows indicate that more evidence may still be needed in this respect. Neurotransmitter dysregulation and hypermelatonemia are collectively referred to as neurochemical abnormalities. ACC, agenesis of the corpus callosum.

In conclusion, hypothalamic dysfunction is considered to be at the heart of SS. Neurochemical abnormalities secondary to hypothalamic dysfunction, such as neurotransmitter dysregulation and hypermelatonemia, play an indispensable role in the onset of SS (Figure 1).

## Is Shapiro syndrome a congenital disorder?

SS is a rare disease. As mentioning above, SS is associated with hypothalamic lesions. Therefore, various factors that cause damage or lesions in the hypothalamus region may lead to SS. We hypothesized that genetic factors, infections, immune diseases, neurodegenerative changes, and et al. are the important etiologies or inducements of SS (Figure 1).

### Genetic factors

ACC is an important clinical feature of SS. According to the California Birth Defects Surveillance Program, the prevalence of ACC is 1.4 per 10,000 live births, and the prevalence of CC dysplasia is 0.4 per 10,000 live births (46). These data suggest that CC abnormalities may be fairly common congenital abnormalities of the central nervous system. ACC involves the partial or complete loss of the main connectivity pathway connecting the two brain hemispheres and may be isolated (without other abnormalities) or complex (co-existing with other abnormalities) (47). In the majority of cases, genetic factors contribute to ACC. These factors include single gene mutations, multiple gene changes, and chromosomal aberrations (48). Belcastro et al. (13) reported a familial SS variant and concluded that SS was an autosomal recessive inheritance pattern. Tambasco et al. (2) also showed that SS was a congenital disorder. Additionally, the neurological channelopathies, which are similar manifestations to SS, have been confirmed as a rare monogenic genetic disease, and a variety of mutant genes, such as SCN, KCNQ, KCNA, CHRNA, GABRB, etc., have been reported (49–52). This also supports that SS may be a genetic disease. However, genetic testing is still unable to identify SS mutations or abnormal genes. Moreover, to date, SS has been reported in men and women aged 2 months to 80 years (13, 28), but only two familial cases (36). Therefore, the role of genetic variation in SS still requires further research in the future.

### Neurodegenerative diseases

PD is the second most common neurodegenerative disorder, affecting about 315 per 100,000 people (53, 54). Patients often develop non-motor autonomic features, such as sleep disturbances, temperature imbalances, pain, cognitive deficits, depression, etc. (54, 55). Recently, it has been reported that patients with PD developed spontaneous periodic hypothermia, and MRI imaging showed normal brain tissue structure, which is consistent with the clinical symptoms of SS (27, 56). These results indicate that PD may be a potential reason of SS. In PD patients with a higher SCOPA-AUT score (PD Autonomic Nerve Outcome Scale), functional connection between HTH and the striatum (caudate nucleus, putamen) and thalamus was significantly reduced, compared with those with a lower SCOPA-AUT score (57). It indicates that thalamo-striatal artery-hypothalamus functional connection is interrupted in PD patients with autonomic nerve dysfunction symptoms. In addition, Renga et al. (56) observed brain tissue sections of PD patients with spontaneous periodic hypothermia and found α -synuclein deposition in the hypothalamus. These results suggest that clinical features of SS occur when PD involves the hypothalamus, and that variants of SS are more common. Furthermore, evidence of hypothalamic involvement has been found in other neurodegenerative diseases. In animal studies, degeneration of the supraspinal nucleus in mice with Alzheimer's disease was detected by magnetic resonance relaxation measurements and immunohistochemical monitoring (58).

### Infections

Nervous system complications caused by infection are common and a hot topic of concern for many researchers. Previous studies have reported a 22-year-old woman who developed encephalopathy after H1N1 influenza, followed by multiple manifestations of hypothalamic dysfunction, insomnia, and persistent PD (59). Gamboa et al. (60) also found antigenic substances associated with neurotropic influenza strain A0 in some neurons of the hypothalamus and substantia nigra in Parkinson's posterior brain. The coronavirus disease 2019 (COVID-19), caused by severe acute respiratory syndrome coronavirus 2 (SARS-CoV-2), has become a global epidemic in recent years (61). Apart from the lungs, the heart and the brain are the main target organs of SARS-CoV-2 (62, 63). SARS-CoV-2 may travel cross the blood-brain barrier or invade the central nervous system *via* the olfactory tract and infect neurons and glial cells that express ACE2, leading to neuroinflammation and neuropathogenesis in brain regions, including the hypothalamus (62–66). COVID-19 has been reported to worsen preexisting symptoms in patients with Shapiro's syndrome variants (67). Additionally, clinical manifestations of SS have been reported in HIV-infected patients (37, 68, 69). Moulignier et al. (68) have demonstrated coordination and consistency between transient hypothermia processes and high levels of HIV replication. Langford et al. (70) found microglial nodules, multinucleated giant cells, perivascular cuff of inflammatory cells, and the presence of HIV-infected cells in the hypothalamic tissue of HIV-infected patients. Collectively, these pieces of evidence suggest that the virus may invade the hypothalamus through various pathways and may eventually lead to SS.

### Immune diseases

Multiple sclerosis (MS) is a chronic, predominantly immune-mediated disease of the central nervous system and one of the most common reasons of neurological disability in young people worldwide (71, 72). The global median prevalence of MS was 33 cases per 100,000 population, with significant differences between countries (71). Prevalence was highest in North America and Europe (140 and 108 cases per 100,000 population, respectively) and lowest in Asia and sub-Saharan Africa (2.2 and 2.1 cases per 100,000 population, respectively) (71, 73). MS usually develops in adults between the ages of 20 and 40 (71). The incidence is about three times higher in women than in men. Paroxysmal hypothermia often occurs in patients with MS (71, 74). Linker et al. (25) reported on a patient with long-term secondary progressive MS and six recurrent episodes of hypothermia as low as 29.9°C with hypotension, bradycardia, coagulopathy, and electrolyte imbalance. MRI showed severe involvement of the CC with associated lesions in the right posterior thalamus (25). These symptoms and MRI findings were consistent with SS syndrome. Toledano et al. (75) demographic analysis of paroxysmal hypothermia in multiple sclerosis found that, among 156 patients, 34 patients had both MS and hypothermia, accounting for 21.8%, but only 4 patients had significant MRI lesions in the hypothalamus. Huitinga et al. (76) found MS lesions in the hypothalamus in 16 out of 17 patients (95%) through the pathological examination of the cadaver, and the overall incidence of active MS lesions was as high as 60%. These data suggest that hypothalamic dysfunction is a potential reason of SS in patients with MS, and lesions in the hypothalamic region are not easily detected by MRI.

## Conclusion

SS is a rare disorder characterized by hypothermia and hyperhidrosis, with or without ACC. SS is a disease with autonomic nerve dysfunction as the main clinical manifestation caused by hypothalamic dysfunction. Genetic, neurodegenerative, infectious and immune diseases are important causes or predisposing factors of SS. There are no reliable imaging or laboratory tests to diagnose SS. The diagnosis of SS by testing neurotransmitter levels in cerebrospinal fluid or serum melatonin levels remains controversial. This review encourages clinicians and investigators to explore more reliable diagnostic methods to improve the diagnosis and treatment rate of SS. Meanwhile, this review recommends that hospitals around the world establish SS registration centers so that clinicians may understand more clinical features of the disease, explore more possible causes of SS, and achieve early prevention, early diagnosis, and early treatment.

## Author contributions

LR designed the study, searched and careened literature, and wrote the manuscript. SY revised the manuscript. MS searched and careened literature. XG and GW designed the study, obtained the funding, and revised the manuscript. All the authors have read and approved the final version of the manuscript.

## Funding

This work was financially supported by grants from the National Natural Science Foundation of China (81972372 to XG) and the Department of Science and Technology of Jilin Province (20190901006JC to GW).

## Conflict of interest

The authors declare that the research was conducted in the absence of any commercial or financial relationships that could be construed as a potential conflict of interest.

## Publisher's note

All claims expressed in this article are solely those of the authors and do not necessarily represent those of their affiliated organizations, or those of the publisher, the editors and the reviewers. Any product that may be evaluated in this article, or claim that may be made by its manufacturer, is not guaranteed or endorsed by the publisher.
